# Theories Informing eHealth Implementation: Systematic Review and Typology Classification

**DOI:** 10.2196/18500

**Published:** 2021-05-31

**Authors:** Milena Heinsch, Jessica Wyllie, Jamie Carlson, Hannah Wells, Campbell Tickner, Frances Kay-Lambkin

**Affiliations:** 1 Centre for Brain and Mental Health Priority Research Centre The University of Newcastle Callaghan Australia; 2 School of Humanities and Social Science The University of Newcastle Callaghan Australia; 3 Newcastle Business School The University of Newcastle Callaghan Australia

**Keywords:** systematic review, eHealth, digital health, mHealth, mobile phone, technology, implementation, adoption, translation, theory

## Abstract

**Background:**

Theory-guided approaches to implementation science have informed translation efforts and the acceptance of eHealth (digital health) interventions in clinical care. However, there is scarce evidence on which theories are best suited to addressing the inherent complexity of eHealth implementation.

**Objective:**

The objectives of this systematic review are to identify theories that inform and explain eHealth implementation and to classify these theories using the typology by Sovacool and Hess for theories of sociotechnical change.

**Methods:**

An electronic search was conducted in the PsycINFO, MEDLINE, Embase, CINAHL, Scopus, Sociological Source Ultimate, Web of Science, ABI/INFORM, EBSCO, and ProQuest databases in June 2019. Studies were included if they were published between 2009 and June 2019; were written in English; reported on empirical research, regardless of study or publication type; reported on one or more theories in the context of eHealth implementation; and were published in a peer-reviewed journal. A total of 2 reviewers independently assessed the titles, abstracts, and full texts. Theories identified were classified using a typology for theories of sociotechnical change, which was considered a useful tool for ordering and analyzing the diverse theoretical approaches as a basis for future theory building.

**Results:**

Of the 13,101 potentially relevant titles, 119 studies were included. The review identified 36 theories used to explain implementation approaches in eHealth. The most commonly used approaches were the Technology Acceptance Model (TAM) (n=33) and the Unified Theory of Acceptance and Use of Technology (UTAUT) (n=32). These theories were primarily concerned with individual and interpersonal elements of eHealth acceptance. Less common were theories that reflect the various disorderly social processes and structural dimensions of implementation, such as the normalization process theory (n=17) and the structuration theory (n=6).

**Conclusions:**

Theories currently informing the implementation of eHealth interventions predominantly focus on predicting or explaining end-user acceptance. Theoretical perspectives that capture the dense and intricate relationships and structures required to enact sustainable change are less well represented in the eHealth literature. Given the growing acknowledgment of the inherent complexity of eHealth implementation, future research should develop and test models that recognize and reflect the multidimensional, dynamic, and relational nature of this process.

## Introduction

In recent years, technological innovation in health care has developed exponentially, and eHealth is now widely viewed as a significant potential contributor to improved quality of care [1,2]. However, despite much policy-level and scholarly discussions of triggering a revolution in health service delivery, problems of implementation and uptake of eHealth among both patients and service providers persist [1,3,4].

Poor uptake of eHealth (a term with contested definitions [5] but, broadly speaking, “health services and information delivered or enhanced through the internet and related technologies” [6]) is often explained in terms of barriers and facilitators [1]. In a recent study, Schreiweis et al [7] identified 77 barriers and 292 facilitators in implementing eHealth services. Similarly, a systematic review by Granja et al [8] identified 27 factors that determine the success or failure of eHealth interventions. Although studies about barriers and facilitators are important, they tend to fall short of capturing the complexity of the implementation process and the multiple *interrelated* factors that determine the translation and uptake of eHealth [1,9].

Evidence suggests that theory-informed approaches to implementation science can enhance the translation and acceptance of eHealth into clinical care [1,10-18]. Theories offer explanatory frameworks and formal heuristic devices that have the potential to move beyond the basic listing of individual facilitators and barriers to implementation, to capture *the dynamic interaction between them* [1]. As Damschroder [19] notes, theory “enables knowledge to emerge out of seeming chaos,” facilitating exploration of complex relationships and interdependencies between variables that unfold in diverse and changing contexts [20]. This is of paramount importance in eHealth settings [1,18], which are characterized by a complicated interplay between patients, clinicians, the health care system, and the eHealth technology.

Many theories and models have been articulated to inform and explain eHealth implementation [15]. Despite this abundance, findings from several reviews show that only a small number of select theories have been used repeatedly across multiple publications and by several authors [21-24]. For example, a recent review by Harst et al [23] of 24 studies of end-user acceptance of telemedicine found that 2 theories accounted for 20 instances of theory use: the technology acceptance model (TAM) and the unified theory of acceptance and use of technology (UTAUT). Similarly, a review on the use of theory in eHealth weight management interventions by Willmott [24] identified 18 studies referencing a theory, of which 16 mentioned either the social cognitive theory or the transtheoretical model.

Theories most commonly used in the literature tend to emphasize individual factors, such as motivation, attitudes, and behavior, rather than the broader social and environmental factors impacting implementation [21,22,25]. This is despite evidence highlighting the multilevel nature of technology implementation in health care and the importance of targeting variables at different levels [1,26]. As Glanz and Bishop [22] noted, social and environmental factors may constrain individuals’ behavior even when they are highly motivated. Therefore, the authors recommend complementing individually oriented theories with theories of social, policy, or organizational change [22].

One hindrance to this is that the current eHealth implementation literature is fragmented across multiple specialty areas and disciplines, making it difficult to locate the range of theories available [27]. To improve the selection and application of theory, it is necessary to identify an array of theories, across diverse disciplines, that have the potential to inform eHealth implementation. A further issue is that many theories contain overlapping constructs but use different terms to describe them [26]. Synthesizing theories according to their similarities would facilitate their selection and application at different levels [27].

To address these issues, we conducted a systematic review and classification of eHealth implementation theories. The review aims to address the following question: “What theories exist across disciplines that have been used to inform or explain eHealth implementation?” Theories identified by our review were classified using the typology by Sovacool and Hess [28] for theories of sociotechnical change. This typology provides an accessible and useful framework for organizing and selecting diverse theoretical options that target variables at different levels. Its use also allows the identification of areas where further theoretical development is required.

## Methods

### Overview

This systematic review was conducted by members of the review team in accordance with the PRISMA (Preferred Reporting Items for Systematic Reviews and Meta-Analyses) guidelines [29]. A PRISMA checklist is available in Multimedia Appendix 1 [29]. The authors adopted a flexible approach by continuing to apply the core principles of systematic review methodology but tailoring the PRISMA guidelines to the needs of this review [30]. As such, formal quality assessment was not conducted for this review, as the perceived validity or trustworthiness of the included studies did not address the overall research question, which sought to identify the existence of theories across a broad and varied body of literature.

### Search Strategy

Electronic searches of PsycINFO, MEDLINE, Embase, CINAHL, Scopus, Sociological Source Ultimate, Web of Science, ABI/INOFORM, EBSCO, and ProQuest Databases were conducted by the review team in June 2019 to identify studies that applied one or more theoretical frameworks to inform eHealth implementation. For this review, implementation was defined as “the scientific study of methods to promote the systematic uptake of research findings and other EBPs (evidence-based practices) into routine practice, and, hence, to improve the quality and effectiveness of health services.” [31]. These databases were chosen because they were deemed to be likely to catalog studies and disciplines relevant to the eHealth context and the specific research question. The search was limited to studies published in the last 10 years (from 2009 to June 2019) and yielded 21,704 abstracts for initial consideration. A full list of key search terms used can be found in Multimedia Appendix 2. All records were converted into an EndNote library and reduced to 13,101 following deduplication. Papers were then title-checked for relevance to the topic and research questions and aims before further screening by 2 independent reviewers (MH and HW) in accordance with the detailed inclusion and exclusion criteria outlined below.

### Eligibility Criteria

Individual studies were included in the review if they were (1) published in the last 10 years (from 2009 to June 2019), (2) published in English, (3) outputs of empirical research or theoretical papers reporting on one or more theories in the context of eHealth implementation (this included all study types and populations), or (4) published in a peer-reviewed journal. Studies were excluded if they were (1) published before 2009, (2) not written in English, (3) studies that did not report on one or more theories applied in the context of eHealth implementation, (4) gray literature not published in a peer-reviewed journal, (5) dissertations, theses, conference proceedings, or abstracts, or (6) any form of literature review. The full eligibility criteria for this review are provided in Textbox 1.

**Eligibility criteria for the review.**

**Inclusion criteria**

- Publication date from 2009 (inclusive) to June 2019
- Australian and international literature in English language
- Papers reporting on one or more theories in the context of eHealth implementation (any study type and population)
- Empirical studies (both quantitative and qualitative)
- Position, discussion, or theoretical papers
- Peer-reviewed articles

**Exclusion criteria**

- Publication before 2009
- Literature in non-English language
- Papers not reporting on one or more theories in the context of eHealth implementation
- Gray literature or not published in a peer-reviewed journal
- Dissertations or theses or conference proceedings or abstracts
- Literature reviews (narrative, scoping, and systematic)

### Identification and Selection of Studies

A total of 2 reviewers (MH and HW) independently applied the predefined inclusion and exclusion criteria to screen for relevant studies from those obtained through database searching. To ensure accuracy, record titles and abstracts were screened manually in EndNote, and documents that did not meet the selection criteria outlined above were excluded by the reviewers. Following 2 rigorous rounds of title and abstract screening, full texts of all potentially eligible studies were examined and further screened by the 2 independent reviewers (MH and HW) using the Covidence web-based software (Veritas Health Innovation Ltd), an effective tool for assisting research teams when performing systematic reviews or meta-analyses [32]. Articles that failed to meet the selection criteria were excluded and then cross-checked to ensure transparency and accuracy surrounding the reasons given for exclusion. Any conflicts in decision making during the screening phase were resolved via discussion between reviewers or, if needed, with the research coordinator (FKL) until consensus was reached.

### Data Extraction and Presentation

As the standardized extraction tool in Covidence did not meet the specific needs of this review, a modified extraction form was developed and piloted by the 2 reviewers (MH and HW) with 10 included studies tabulated and refined accordingly. The modified extraction form was tailored to include characteristics relevant to the research question. The characteristics extracted by the reviewers included (1) name of theory, (2) description, (3) instances of theory use, (4) examples of theory application, and (5) theory type. Instances of theory use refer to the number of occurrences in which a theory was used. As several studies used more than one theory, the total number of theory instances exceeded the number of papers included in the review. Examples of theory application were drawn from the literature to specify how each theory informed eHealth implementation. The reviewers then determined each theory type by drawing on the typology by Sovacool and Hess [28] for theories of sociotechnical change. This typology categorizes theories according to where they tend to *center* their analysis. The term *center* is intended to convey that a theory may involve elements of multiple types but that it approximates one ideal type above all. This typology was considered a useful tool for ordering and analyzing the diverse theoretical approaches identified, as a basis for future theory building [33].

The typology includes 5 categories: agency, structure, relations, meaning, and norms. *Agency-centered* theories relate to people’s individual actions, beliefs, and attitudes, and assume that these can be explained without deeper consideration of broader social and systemic elements [28,34,35]. In contrast, *structural* theories propose that people are influenced largely by external forces beyond their control, such as their organizational, political, or macrosocial environments [28,35]. *Relational* theories attempt to interpret the interactional processes that influence the circulation of knowledge throughout different social networks. They view technology and society as coproduced and coconstructed, with no single dimension creating change by itself [28,36]. *Meaning-centered* theories focus on language, ideas, symbolism, narratives, rhetorical visions, and other cognitive dimensions that both orient action and are changed by it. *Normative* theories offer criteria by which to assess the positive or negative impact of technology on society or on a specific group. A sixth category, *combined* theories, was added to these 5 categories. This included meta-theories that explored a combination of individual, structural, or relational frameworks. All authors (MH, JW, JC, HW, CT, and FKL) reviewed and agreed upon the classification of theories using this typology.

## Results

### Search Results

**Figure 1:**
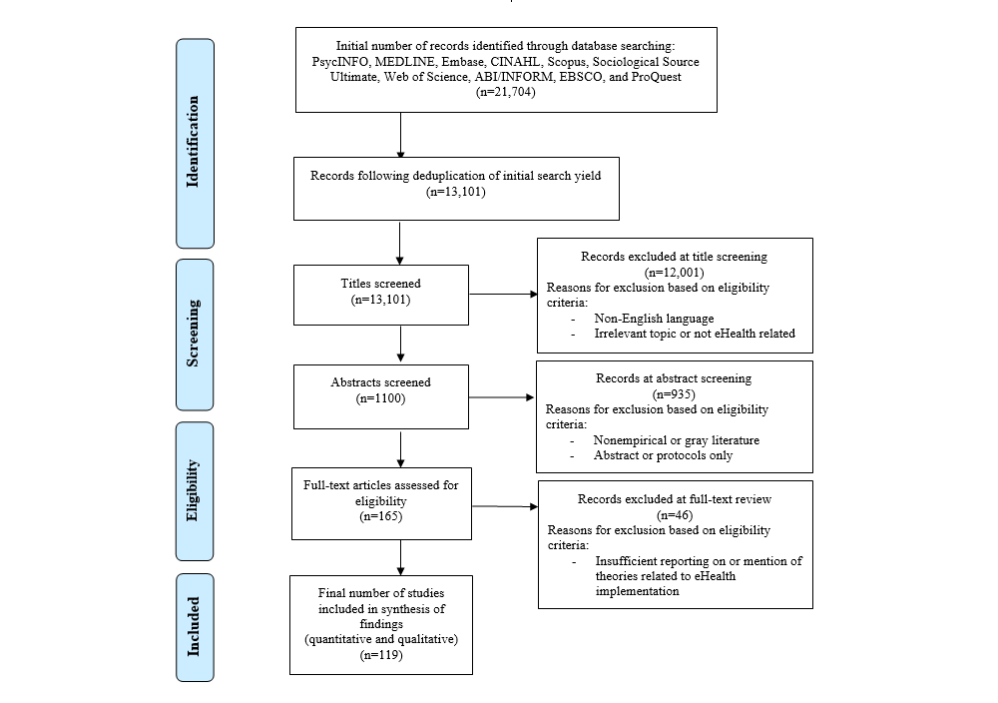
Flowchart of studies included and excluded from the systematic review.

The electronic search of key databases resulted in 21,704 potentially eligible articles (Figure 1). This number was reduced to 13,101, following deduplication. Of these, 12,001 papers were excluded based on title screening and application of the eligibility criteria previously outlined. Key reasons for exclusion of papers at title screening included eliminating those that were in non-English language or those that reported on an irrelevant topic to the research question, for example, non-eHealth or theory-related papers. The abstracts of the remaining 1100 papers were then independently screened by reapplying the inclusion and exclusion criteria, and a further 935 papers were excluded. Key reasons for exclusion at abstract screening included nonempirical or gray literature and papers that reported abstracts or protocols only. Following a full-text review of the remaining 165 articles, an additional 46 articles were excluded because of insufficient reporting on or mention of theories related to eHealth implementation. In total, 119 articles met the full, predefined eligibility criteria and were included for data extraction and synthesis of findings. The PRISMA flowchart in Figure 1 details the process of eligibility and study selection.

### Theory Summary and Classification

The summary (including theory name, description, instances of theory use, and examples of application to implementation) and classification of all theories used to inform and explain eHealth implementation is provided in Table 1. In total, 36 distinct theories were identified. Classification of these theories using the typology by Sovacool and Hess [28] showed that the theories used in the literature were predominantly agency centered (19/36, 53%), followed by relational (7/36, 20%), structural (6/36, 16%), meaning (3/36, 8%), and combined theory types (1/36, 4%). No normative theories were identified.

**Table 1:** Summary and classification of eHealth implementation theories.

Theory	Description	Instances of theory use	Examples of theory application	Type
TAM^a^	Proposes that technology acceptance and use are affected by an individual’s perceived ease of use, perceived usefulness, and subjective norms	33	“This study tested an extended version of TAM and used this to explain the attitude of nursing staff towards using electronic patient record. The addition of external variables was shown to increase the predictive value of the model.” (de Veer and Francke, 2010) [37] “Our research introduces new variables to the TAM model in order to suit this particular study. These new variables include staff information technology experience, technical infrastructures, security concerns, and information sharing. These additional independent factors enhance the TAM’s predictive power.” (Zayyad and Toycan, 2018) [38]	Agency centered
UTAUT^b^	Proposes that behavioral intention to use eHealth interventions is affected by individual effort expectancy, performance expectancy, social influence, facilitating conditions, and habit	32	“We extended the UTAUT model to investigate further context-related predictors of acceptance and postulated that eHealth literacy, which means the ability to find, evaluate, and utilize internet-based health information to health problems, and knowledge of and experience with eHealth interventions were positively related with eHealth acceptance, based on previous evidence.” (Henneman et al, 2017) [39] “To assess prospects for broad adoption of Electronic Integrated Antenatal Care, we trained midwives in the use of the system and then used the UTAUT survey to assess their intention to adopt the tool.” (Markam et al, 2018) [40]	Agency centered
NPT^c^	Explains the social processes (eg, coherence, participation, collective action, and reflexive monitoring) through which eHealth interventions are operationalized	17	“Deductive thematic and content analyses were undertaken by two independent coders [on data derived from semistructured interviews]. The NPT coding framework was used...A coding protocol was developed, trialled and refined using an additional transcript from each network.” (Bagot et al, 2017) [41] “In this paper we present a simplified set of 16 statements that express key elements of NPT, but which can be applied without a detailed knowledge of the underlying theory...we sought to better understand the ways that potential users of NPT could apply it to real world problems. Between 2006 and 2009 we engaged with multiple potential users. Engaging potential users included presentations to researchers and practitioners that linked NPT’s core constructs to practical research and development problems” (May et al, 2011) [42]	Relational
DOI^d^ theory	Explains how an eHealth innovation gains momentum and diffuses through a specific population. This process is affected by the innovation itself, time, channels of communication, and an individual’s social system	16	“The semistructured interview built upon Rogers’ Diffusion Theory and examined the five general stages of diffusion (knowledge, persuasion, decision, implementation, and confirmation) that occurred during the clinic development. We asked respondents to describe the local mental health services before telehealth was introduced; the process by which the telehealth was introduced to—and adopted by—their organization; and the acceptance of telehealth by the community.” (Brooks et al, 2012) [43] “We used Rogers’ DOI theory framework for how new innovations are adopted by organizations and Greenhalgh’s subsequent work adapting the framework for health care settings. We used these frameworks to deductively explore factors that might help intervention better diffuse in each clinic setting...The analysis mapped themes identified in the qualitative data to the DOI framework described above.” (Lin et al, 2016) [44]	Agency centered
Structuration theory	Models the relationship between agency and structure. In eHealth implementation, interventions are configured and coconstructed over time and can be adapted or revised to better accommodate different settings and needs	6	“This study represents an empirical example of how AST^e^ coupled with literature on organizational change offer a better understanding of technology implementation practices. Our findings complement past AST research, claiming that implementation success and users’ change attitudes are two important outcomes associated with appropriation.” (Barrett and Stephens, 2017) [45]	Relational
CFIR^f^	A meta-theoretical framework that provides an overarching typology of constructs relating to implementation, including intervention characteristics, outer setting, inner setting, and individual characteristics	6	“The main results presented subsequently address identified barriers and facilitators influencing the implementation of internet-based patient-provider communication in 5 hospital units using CFIR to identify determinants distinguishing between high and low implementation success.” (Varsi et al, 2015) [46] “Semistructured interviews were developed based on the constructs of the CFIR which provides a pragmatic organization of theory-informed constructs known to impact implementation success across five domains...Interview transcripts were analyzed by two independent investigators using the Framework Method. This involved a largely deductive thematic analysis using a codebook based on the constructs of the CFIR.” (Ware et al, 2018) [47]	Combined
ANT^g^	Posits that objects have agency and that a combination of network components (both human and nonhuman) helps create and/or influence social effects (such as implementation)	4	“This is a qualitative study, in which ANT was used as a theoretical reference. The ANT proposes to follow and map actant’ movements and the influences traversing their reciprocal connections...Our methodological reference was the ‘cartography of the controversies,’ considered a set of techniques to explore and visualize controversies and discussions, observing and describing social debate, especially—but not exclusively—around technical and scientific problems.” (Cavalcante et al, 2019) [48]	Relational
TPB^h^	Extends the theory of reasoned action to incorporate actors’ perceived control over the outcomes of their behaviors	3	“This study integrated the Technology Acceptance Model and TPB frameworks to evaluate patient acceptance of e-health services. The Technology Acceptance Model and TPB frameworks were developed based on the theory of reasoned action.” (Albar and Hoque, 2019) [49]	Agency centered
Institutional theory	Posits that an organization’s environment is capable of strongly influencing the development, acceptance, and use of eHealth interventions	3	“Due to the unique and highly institutionalized health-care environment in the US, we therefore focus on EHR^i^ adoption as an isomorphic institutional change that leads to the decision to acquire and make available electronic records for use in ambulatory services...we believe that institutional theory is a useful framework for analyzing EHR adoption.” (Sherer et al, 2016 [50])	Structural
SCT^j^	Posits that the acquisition of new knowledge and perceived self-efficacy in using interventions is influenced by observing others in the context of social interactions and experiences (including the media)	3	“The research framework encompasses three categories. The proposed bidirectional interaction between the belief types associated to these categories is based on SCT’s assumption that individual behaviour is shaped by outcome expectations (behavioural factors), self-perception (personal factors) and the social and physical environment (environmental factors).” (Weeger and Gewald, 2015) [51]	Agency centered
Theory of reasoned action	A social-psychological attitude-behavior model that examines normative social influences on behavioral intention	2	“The theoretical background of this study focuses on the Theory of Reasoned Action...to analyze the influence of smartphones at tertiary hospitals. This study also applies the Technology Acceptance Model...Based on the accumulated knowledge from Kim and Chang, and Chang, the research model was derived...” (Moon and Chang, 2014) [52]	Agency centered
IT^k^	Posits that the identity associated with a particular role is likely to drive individual decision making about eHealth interventions	2	“We briefly reviewed the existing state of research on health IT adoption in information science and the medical informatics literatures. This is followed by a discussion of identity theories and their application to unique aspects of the healthcare context.” (Mishra et al, 2012) [53]	Agency centered
PMT^l^	Explains how the perceived threat of a new eHealth intervention affects individual adoption and implementation attitudes and behaviors	2	“To study the influence of PMT components on users’ mobile health adopt intentions, we developed an integrated model based on the PMT theory and the moderating role of gender, age, and education.” (Guo et al, 2015) [54]	Agency centered
Health belief model	Suggests that a personal threat, together with belief in the effectiveness of the proposed behavior, predicts the likelihood of engaging in that behavior	2	“We applied a new adoption model that combines 3 different theories, namely, extended unified theory of acceptance and use of technology, health belief model, and the diffusion of innovation; all the 3 theories provided relevant contributions for the understanding of EHR portals. To test the research model, we used the partial least squares causal modelling approach.” (Tavares and Oliveira, 2018) [55]	Agency centered
BMHSU^m^	A model for predicting and explaining factors that lead to the use of eHealth services. These can include predisposing, enabling, and needs factors	2	“We used a multiphase, longitudinal study design. The four objectives were addressed in the four proposed stages of use. Applying Venkatesh’s UTAUT and Anderson’s BMHSU, we conceptualized that caregivers will go through the stages of consideration, initiation, utilization, and outcomes.” (Chiu and Eysenbach, 2010) [56]	Agency centered
Sociotechnical systems theory	Proposes an interdependent relationship between the social and technical aspects of an organization (eg, human-computer interface). It suggests that these must be viewed congruently to optimize the implementation of new eHealth interventions	1	“Drawing insight from the theoretical lens of Sociotechnical theory, the seven clusters of factors required for health-risk assessments implementation could be read as belonging to three overarching aspects: Technical (cluster 1, 2 and 3), Social-Patient (cluster 4 and 5), and Social-Provider (cluster 6 and 7).” (Ahmad et al, 2012) [57]	Relational
ECT^n^	Posits that expectations, coupled with perceived performance of an eHealth intervention, lead to postpurchase satisfaction	1	“The conceptual framework of this research was cited from ECT. This theory was adopted extensively in the field of Marketing and Management Information System since the 1980s, though rarely applied to the healthcare field. We also used all the constructs derived from Oliver’s ECT model in our research to present the original concept of this model.” (Chou et al, 2012) [58]	Meaning centered
PCT^o^	Posits that past experiences of using eHealth technologies and individual assumptions in relation to the design, impact, ownership, and value of these technologies can strongly influence acceptance or reluctance toward an eHealth intervention	1	“Arguably, PCT is a very useful framework for making more visible what lies below the surface of human problems in organisations...Consequently, this paper employs PCT as a theoretical lens to understand clinicians’ reluctance to accept and use new IT systems in the NHS.” (Fernando et al, 2012) [59]	Meaning centered
Resource dependence theory	Asserts that acquiring and maintaining resources (eg, eHealth interventions) is key to organizational survival. Scarce resource availability or uncertainty about the environment motivates managers to act in ways to secure more resources and reduce their uncertainty	1	“This study used the resource dependence theory to understand how the environment influences hospitals’ investments in health information technology.” (Tarver and Menachemi, 2018) [60]	Structural
Theory of middle managers’ role	Hypothesizes that middle managers promote implementation by fulfilling 4 roles: diffusing information, synthesizing information, mediating between strategy and day-to-day activities, and selling intervention implementation	1	“Although the theory has received some empirical support, the extent to which it aligns with middle managers’ experience in practice is unclear. The objectives of this study were to (1) assess alignment between middle managers’ experience and the theory’s hypothesized roles and activities and (2) elaborate on the theory with examples from middle managers’ experience.” (Birken et al, 2016) [61]	Structural
PAD^p^ emotional state theory	Asserts that all emotional responses to physical and social environmental stimuli can be captured in 3 dimensions: pleasure (enjoyment), arousal (alertness), and dominance (control), which subsequently inﬂuence human behavior	1	“It is the first attempt at integrating UTAUT and PAD theories to account for cognitive and affective factors in explaining technology adoption. The theory enhances the theoretical base of technical communication research by enabling theory-driven design and development of wireless health communication systems.” (Alaiad and Zhou, 2017) [62]	Agency centered
Information behavior theory	Posits that information systems serve as a bridge between users and information resources. They consist of mediators (people who help users seek information and share the same social norms) and technologies (techniques and tools) that help users with the search	1	“In this study, a critical inquiry approach was used to theorize usage behaviour through an analytic integration of three theoretical models. In our model, the driving question was as follows: What usage behaviour can be explained by Anderson’s BMHSU, Venkatesh’s UTAUT, and Wilson’s and Chatman’s information behaviour theories? We answered this question by constructing a concept map that integrates the theoretical and empirical findings. The concept map and five sub-themes that influence usage and non-usage behaviour will be reported.” (Chiu and Eysenbach, 2011) [63]	Agency centered
JDRM^q^	Suggests that strain is a response to imbalance between demands on individuals and the resources they have to deal with these demands	1	“To answer our research questions, we took guidance from 2 theoretical models to ultimately derive the model...Based on the JDRM and UTAUT, we hypothesized that each of the 4 factors will positively impact provider satisfaction, and inversely relate to intention to quit.” (Hysong et al, 2014) [64]	Agency centered
Cultural dimension theory	Shows the effects of a society’s culture on the values of its members and how these values relate to implementation behaviors	1	“To explore the influence of culture on e-health adoption, both TAM and Hofstede’s cultural dimension model are incorporated in this study.” (Hoque and Bao, 2015) [65] “The second section [of the survey questionnaire] consists of subject perception of each variable in the model. The measurement items were adopted from prior research and modified based on the e-health context in Bangladesh.” (Hoque and Bao, 2015) [65]	Meaning centered
Affect theory	Claims that there are 3 primary types of affect or emotion, including positive (joy, interest, and excitement), neutral (surprise), and negative (anger, terror, and disgust). These affective states may be advantageous or disadvantageous in users’ acceptance of new information systems	1	“Grounded in current theories of affect this study examines the role positive and negative moods play on the acceptance of a specialized telemedicine system for microbiology consultation and diagnostics, referred to as telepathology.” (Djamasbi et al, 2009) [66] “Using cognitive theories of affect, we propose an extension to TAM by arguing that users’ affect plays a significant role in influencing their attitude towards a new healthcare information system.” (Djamasbi et al, 2009) [67]	Agency centered
Activity theory	Focuses on understanding the mental capabilities of individuals by analyzing the cultural and technical aspects of human actions	1	“We propose an integrated research model for exploring and understanding critical factors influencing physicians’ intention to use computerized Clinical Practice Guidelines by incorporating activity theory (three dimensions of factors) with TAM concepts (intention as dependent variable).” (Hsiao and Chen, 2016) [68]	Agency centered
Social capital theory	Contends that social relationships are resources that can lead to the development and accumulation of human capital	1	“This study integrated social capital theory, social cognitive theory, and TAM to develop a comprehensive behavioral model for analyzing the relationships among social capital factors (social capital theory), technological factors (TAM), and system self-efficacy (social cognitive theory) in telehealth.” (Tsai, 2014) [69]	Agency centered
Contingency theory	Claims that there is no best way to organize or lead an organization or to make decisions. Instead, the optimal course of action is dependent upon the internal and external situation	1	“In this study, we use contingency theory as a base to hypothesize how contingent factors, above and beyond traditionally considered ‘dominant’ factors often associated with supply-side adoption, may affect the adoption of patient portals by ambulatory-care clinics.” (Baird et al, 2012) [70]	Structural
Social information processing theory	Predicts that technology-related attitudes and behaviors are not individually laden but socially constructed	1	“When relating social information processing theory and the social influence model to organizational change situations, we see that both theories provide a framework for understanding previous scholars’ arguments advocating the noteworthy role informal, coworker communication plays in effective organizational and healthcare change.” (Barrett and Stephens, 2017) [71]	Relational
Consequence of modernity	Suggests that use of technologies is influenced by trust and sense of security in the absence of complete information from face-to-face interactions	1	“Our research uses concepts from Giddens’s structuration theory and consequence of modernity to understand clinical users view on telehealth service when first introduced in their work setting.” (Sharma et al, 2010) [70]	Agency centered
Social worlds theory	Proposes that social worlds are self-organizing units in which people share resources, information, and assumptions about what is important and ideas about what types of activities are desirable	1	“Using the notions of social worlds, trajectories, and boundary objects enables us to show how mobile information technology innovation in Danish home care can facilitate negotiation and collaboration across different social worlds in one setting while becoming a source of tension and conflicts in others.” (Nielsen and Mengiste, 2014) [72]	Relational
Boundary objects	Boundary objects serve as interfaces between multiple social worlds and facilitate the interaction; communication; and flow of information, concepts, skills, and materials between diverse social actors	1	“This article contributes to this emerging research domain by using notions of social worlds, trajectories, and boundary objects and applying these constructs in an empirical investigation in Danish elderly home care. Our discussion therefore focuses on two key issues: to what extent different interests among multiple social worlds have been negotiated in the trajectory of adopting and diffusing mobile IT and to what extent boundary objects have aligned the interests of stakeholders from different social worlds.” (Nielsen and Mengiste, 2014) [72]	Relational
Adult learning theory	Posits that one’s learning context influences learning outcomes. Thus, knowledge evolves not only through formal learning activities, such as training programs, but also through the context and culture in which they are delivered	1	“We drew on concepts from social cognitive theory and situated cognition theory (from adult learning theory) to frame our study of training practices within the ambulatory EHR system implementation process. These theories helped us develop five propositions related to the importance of training in promoting meaningful use of EHR systems.” (McAlearney et al, 2012) [73]	Agency centered
Social contagion theory	A theory of collective behavior that explains how ideas and opinions spread in a social network. It holds that actors’ behaviors are a function of their exposure to others’ behaviors	1	“Based on extensive literature review and drawing upon two theories—social contagion theory and task technology fit theory—I argue that the adoption of EHR system is contagious among health care providers; however, the contagion effect depends on the fit between the characteristics of EHR system and the characteristics of health care providers.” (Gan, 2015) [74]	Agency centered
TTF^r^ theory	Explains how technology interacts with the tasks or activities of an organization and impacts their performance	1	“The findings also suggest that the two factors (TTF and social contagion) are not independent and the interaction of them plays a more important role that either of them alone.” (Gan, 2015) [74]	Structural
Technology organization environment theory	Predicts that technology adoption is influenced by factors relating to technological, organizational, and environmental dimensions	1	“To investigate the factors influencing the adoption of HIS^s^ in the hospitals’ work processes, this study proposed the initial theoretical framework based on the combined Technology Organization Environment, institutional theory, and Human Organization Technology fit model.” (Ahmadi et al, 2017) [75]	Structural

^a^TAM: technology acceptance model.

^b^UTAUT: unified theory of acceptance and use of technology.

^c^NPT: normalization process theory.

^d^DOI: diffusion of innovations.

^e^AST: adaptive structuration theory.

^f^CFIR: Consolidated Framework for Implementation Research.

^g^ANT: actor-network theory.

^h^TPB: theory of planned behavior.

^i^EHR: electronical health record.

^j^SCT: social cognitive theory.

^k^IT: identity theory.

^l^PMT: protection motivation theory.

^m^BMHSU: behavioral model of health service utilization.

^n^ECT: expectation confirmation theory.

^o^PCT: personal construct theory.

^p^PAD: pleasure, arousal and dominance.

^q^JDRM: job demands resource model.

^r^TTF: Task Technology Fit.

^s^HIS: hospital information system.

In total, 53% (19/36) of theories were classified as *agency centered*. Individual theories that occurred most frequently in the literature were the TAM by Davis and Venkatesh [76] (33 instances), UTAUT by Venkatesh [77] (32 instances), and Diffusion of Innovations Theory by Rogers [78] (16 instances). These theories were found to be primarily concerned with the individual and interpersonal elements of eHealth implementation. Although they did, to some extent, appear to consider the influence of organizational and social factors on eHealth adoption, individual attitudes, behaviors, and motivations remained the core focus of theoretical analysis. Theories classified as individual examined the adoption of eHealth either before or soon after the implementation of an intervention. However, they did not emphasize any form of user involvement in the development of an intervention. These theories tended to depict adoption as a temporally discrete and relatively immediate event, rather than as one stage in a larger multistage process. They often focused on what people were going to do soon, a decision they are about to make, or a behavior they need to alter. The diffusion of innovations theory provides an exception, as this theory considers time to be an essential factor influencing adoption [79].

A total of 20% (7/36) of theories identified in the literature were classified as *relational*. Of these, the normalization process theory (NPT) by May et al [80] occurred most frequently in the literature (17 instances), followed by structuration theory (ST) [81] (6 instances) and actor-network theory (ANT) [82,83] (4 instances). Sociotechnical systems theory, social information processing theory, social worlds theory, and boundary object theory occurred only once each in the literature. Relational theories emphasize social relations and interactions at the human-technology interface. They highlighted the complex networks of social structure and meaning in which people are embedded, proposing that the translation of knowledge is facilitated by processes of circulation both within and across different social worlds. Some relational theories, such as ANT and ST, emphasized the role of nonhuman actors, such as computer software or programs, in transforming and mediating social relationships. These theories tended to view technology and society as coconstructed or coproduced, with no single dimension dictating change by itself. Within these theories, coproduction and implementation were often described as continuous processes, in which eHealth interventions were adapted to better accommodate different end-user settings and needs.

A total of 16% (6/36) of theories were classified as *structural*. The most common structural theory was institutional theory (IT) [84] (3 instances). Resource dependence theory, theory of middle managers’ role, contingency theory, task technology fit theory, and technology organization environment theory occurred only once each in the literature. These theories conceptualized *structure* as including institutional or organizational systems as well as political, cultural, and other macrosocial environments. They often assumed that people are constrained or influenced by external forces frequently beyond their comprehension or control. For example, IT posits that organizational structures and cultural norms drive eHealth implementation, despite strong political influence.

A total of 8% (3/36) of theories were classified as *meaning centered*: expectation confirmation theory, personal construct theory, and cultural dimension theory. Each of these theories occurred only once in the literature. These theories tended to focus on the cognitive dimensions (expectations, perceptions, and beliefs) that explain people’s willingness to accept the use of new health technologies. Although some meaning-centered theories, such as cultural dimension theory, have considered the influence of cultural values on the adoption and use of eHealth, these theories nonetheless centered their analysis at the individual level and were often used in combination with agency-level theories.

The Consolidated Framework for Implementation Research (CFIR) [85] was the only theory to be classified as a *combined* theory type. This theory is a meta-theoretical framework that provides a comprehensive listing of individual, social, and organizational constructs thought to influence eHealth implementation. However, it does not consider how these factors might be interrelated or how changes occur.

## Discussion

### Principal Findings

Evidence from a range of disciplines suggests that theory-informed approaches to implementation science are integral to the translation and implementation of eHealth into clinical care [1,10-18]. Analysis of the 119 studies included in this review identified 36 distinct theories that inform or explain eHealth implementation. However, only a few selected theories (UTAUT and TAM) were dominant, which is consistent with the findings from previous reviews [21-24]. Although these theories have been empirically proven to explain or predict certain aspects of implementation, Willmott et al [24] and Davis et al [21] caution that overreliance on *common* or *favorite* theories without direct questioning of their underlying assumptions limits progress in the field.

The typology by Sovacool and Hess [28] facilitated a closer examination of the assumptions underlying eHealth implementation theories. The findings revealed that the majority of theories were agency centered, emphasizing individual factors rather than the broader social and environmental factors impacting implementation. Although these findings were consistent with previous reviews [21,22,24,86], the wider net cast for this review provided the needed validation that this trend can be observed across multiple specialty areas and disciplines [27]. This calls into question whether theories currently being used to inform and explain the eHealth implementation adequately address the multiple and complex factors that influence the implementation process, and highlights the need for more dynamic, multilevel models of eHealth implementation [21,23,87].

This review identified a number of theories classified as relational or structural, which, to varying degrees, capture the complexity and multilevel nature of eHealth implementation. The most commonly cited relational theories were NPT, ST, and ANT. These theories recognize the important role of actors, relationships, and networks in mobilizing knowledge and embedding interventions into everyday practice. For ANT, networks are made up of both human and nonhuman *actors*, and technologies are understood to have agency and the potential to transform human interactions [88,89]. From this perspective, it may be a particularly useful theory for examining the implementation of eHealth technologies and the impacts these technologies have on human behavior. A criticism of ANT is that it has a *flat* ontology and refuses to consider institutional sources of power and inequality. Here, NPT and ST offer a possible extension, as both theories recognize the inseparable intersection between individual agents and wider social and organizational structures and norms. Structural theories also consider the influence of external forces on individual behavior and decision making. For example, IT, the most commonly used structural theory in this review, posits that an organization’s environment is capable of strongly influencing the development, acceptance, and use of eHealth interventions. This theory is considered particularly relevant for application in eHealth environments, which are highly institutionalized and subject to multiple regulatory forces, high levels of professionalism, and growing network externalities that can influence adoption decisions [50].

Of particular interest was the lacuna of normative theories identified in this review. Normative theories attempt to answer whether a technology is a net positive or negative for society and individuals [28]. To do so, they often rely on evaluative criteria determined by ethics, moral studies, political ecology, or social justice. Social justice theory and sustainable development are 2 common examples of normative theories. The absence of normative theories in eHealth implementation studies is emblematic of the broader tendency of implementation science to overlook the importance of contextual factors, such as economic, social, historical, and political forces, that perpetuate inequalities in the delivery of health care services [90]. This omission is concerning in the context of eHealth, as digital technologies have been found to exacerbate inequalities associated with older age, lower level of educational attainment, and lower socioeconomic status [91]. Future research should not shy away from normative questions of equity, justice, and sustainability and should find ways to incorporate theoretical approaches that enable exactly that.

When incorporating or combining theories, Sovacool and Hess [28] highlight the need for careful consideration of the *epistemological baggage* of different approaches. Combining multiple theoretical approaches may offer a more complete understanding or explanation, yet such combinations may mask contrasting assumptions regarding key issues [92]. For instance, are people driven primarily by their individual attitudes and motivation or do pervasive organizational cultures and social systems impose norms and values that shape people’s behavior, making individual characteristics relatively unimportant? These challenges may account for the tendency of theories to target variables at the same level. One exception was the CFIR framework, which was the sole theory that provided a *menu* of constructs at different levels for researchers to choose from. However, although CFIR recognizes the multilevel nature of eHealth implementation, it does not consider the relationship between constructs or how change takes place, leading Nilsen [92] to contend that it should not be considered a theory at all. Further research is needed to explore how diverse theoretical perspectives can be brought together in ways that capture the dynamic interaction between constructs [1], while avoiding disconnects and incompatibilities [28].

### Limitations

This study has several limitations. First, papers not published in English were excluded, which may indicate a selection bias. The decision to keep the research question and inclusion criteria for this review broad resulted in a high yield of papers and, to some extent, reduced the specificity of search results. This decision was made to ensure the identification of the full spectrum of theories being used to inform and explain eHealth implementation. Restriction of inclusion criteria in previous systematic reviews [24] led to the omission of a number of key theories that provide a more comprehensive explanation of the various constituents of the implementation processes. A further limitation is that the protocol for this systematic review was not registered. However, every care was taken to ensure compliance with the core principles of the systematic review methodology. As Mallett [30] noted, systematic reviews do not constitute a homogenous approach, and researchers may adopt a more flexible approach that better suits their research purpose while continuing to comply with the principles for conducting a systematic review. Finally, the literature search for this review was conducted in June 2019. Given the rapid rate of publication in the field of eHealth, it is likely that recent relevant articles have not been included. As completing an updated search was not feasible for the research team, we suggest that future studies must continue to identify theories used to inform and explain the implementation of eHealth interventions.

### Conclusions

This systematic review identified 36 theories that are being used to inform and explain eHealth implementation and classified these theories using the categories adapted from the typology by Sovacool and Hess [28] for theories of sociotechnical change. The results highlight the dominance of theories that focus mainly on individual readiness to accept health technologies rather than the various disorderly social processes or systemic dimensions of implementation. This calls into question whether theories currently being used to inform and explain eHealth implementation adequately address the multiple and multilevel factors that influence the implementation process. Nonetheless, this review identified a number of theories classified as relational, structural, or combined, which, to varying degrees, capture the complex interactions within a wider organization and policy system. Although less prominent in the literature, these theories may be particularly applicable to the implementation of eHealth in health settings and services.

## Appendix

Multimedia Appendix 1 PRISMA (Preferred Reporting Items for Systematic Reviews and Meta-Analyses) checklist.

Multimedia Appendix 2 Key search terms used.

## Abbreviations

ANT: actor-network theory
CFIR: Consolidated Framework for Implementation Research
eCliPSE: Electronic Clinical Pathways to Service Excellence
IT: institutional theory
NPT: normalization process theory
PRISMA: Preferred Reporting Items for Systematic Reviews and Meta-Analyses
ST: structuration theory
TAM: technology acceptance model
UTAUT: unified theory of acceptance and use of technology
