# Supplementary material for: Theories Informing eHealth Implementation: Systematic Review and Typology Classification
Source: J Med Internet Res. 2021 May 31;23(5):e18500. doi: 10.2196/18500 (PMC8204232; doi:10.2196/18500)
Supplement: Multimedia Appendix 2 [file jmir_v23i5e18500_app2.docx]

**Multimedia Appendix 2:** Key search terms used

| **eHealth/Technology** | **Intervention** | **Theory** | **Implementation** | **Mental health** |
| --- | --- | --- | --- | --- |
| “eHealth” | “intervention” | “theory” | “implement” | “mental health” |
| “mhealth” | “program” | “framework” | “disseminate” | “mental illness” |
| “mobile health” | “therapy” | “model” | “adopt” | “mental disorder” |
| “electronic health” | “treatment” |  | “uptake” | “health care delivery” |
| “eMental health” | “education” |  | “diffuse” | “health care services” |
| “ePublic health” | “counselling” |  | “transform” | “mental health services” |
| “telemedicine” | “help” |  | “translate” | “healthcare” |
| “telehealth” | “assistance” |  | “transform” |  |
| “telepsychology” | “support” |  | “transfer” |  |
| “teletherapy” | “instruct” |  | “utilise” |  |
| “internet” | “train” |  | “integrate” |  |
| “phone” | “advise” |  | “scale up” |  |
| “telephone” | “manage” |  | “incorporate” |  |
| “smartphone” | “deliver” |  |  |  |
| “cellphone” | “direct” |  |  |  |
| “computer” | “approach” |  |  |  |
| “online” | “involve” |  |  |  |
| “web” | “participate” |  |  |  |
| “apps” | “promote” |  |  |  |
| “digital” | “teach” |  |  |  |
| “text message” | “lead” |  |  |  |
| “Facebook” | “deliver” |  |  |  |
| “social media” | “service” |  |  |  |
| “email” | “new technology” |  |  |  |
| “Twitter” | “information technology” |  |  |  |
| “sms” |  |  |  |  |
| “mms” |  |  |  |  |
| “blog” |  |  |  |  |
| “chatroom” |  |  |  |  |
